# Injection of Lidocaine Alone versus Lidocaine plus Dexmedetomidine in Impacted Third Molar Extraction Surgery, a Double-Blind Randomized Control Trial for Postoperative Pain Evaluation

**DOI:** 10.1155/2021/6623792

**Published:** 2021-01-25

**Authors:** Javad Alizargar, Milad Etemadi Sh, Nasser Kaviani, Shu-Fang Vivienne Wu, Keyvan Jafarzadeh, Parisa Ranjbarian, Nan-Chen Hsieh

**Affiliations:** ^1^Research Center for Healthcare Industry Innovation, National Taipei University of Nursing and Health Sciences, Taipei City 112, Taiwan; ^2^Department of Oral and Maxillofacial Surgery, Dental Implants Research Center, Dental Research Institute, School of Dentistry, Isfahan University of Medical Sciences, Isfahan, Iran; ^3^Department of Oral and Maxillofacial Surgery, Isfahan University of Medical Sciences, Isfahan, Iran; ^4^College of Nursing, School of Nursing, National Taipei University of Nursing and Health Sciences, Taipei City 112, Taiwan; ^5^Isfahan University of Medical Sciences, Isfahan, Iran; ^6^Department of Endodontics, Isfahan (khorasgan) Branch, Islamic Azad University, Isfahan, Iran; ^7^Department of Information Management, National Taipei University of Nursing and Health Sciences, Taipei City 112, Taiwan

## Abstract

**Objectives:**

Administration of medications such as dexmedetomidine as a topical anesthetic has been suggested in the pain control in dentistry. This double-blind randomized control trial study evaluated postoperative pain and associated factors following impacted third molar extraction surgery. Lidocaine alone was taken as the control and lidocaine plus dexmedetomidine as the intervention.

**Materials and Methods:**

Forty patients undergoing mandibular third molar extraction entered the study and were randomly allocated to the control and interventional groups. 0.15 ml of dexmedetomidine was added to each lidocaine cartridge and the drug concentration was adjusted to 15 *μ*g for the intervention group while only lidocaine was used in the control group. A visual analog scale was used to measure and record pain levels at the end of the surgery and 6, 12, and 24 hours after the surgery and number of painkillers taken by the patients after the surgery was also recorded.

**Results:**

Pain scores of the intervention group decreased significantly during the surgery and also 6, 12, and 24 hours after the surgery compared to the control group. The pain score was correlated significantly with our intervention during the surgery and also 6 and 12 hours after that (all *P* value < 0.05). There was a nonsignificant reduction in the number of painkillers taken by the patients at 6, 12, and 24 hours after surgery (all *P* value > 0.05).

**Conclusion:**

In patients undergoing molar surgery, administration of a combination of dexmedetomidine and lidocaine is beneficial for the pain control. *Clinical Relevance.* Compared to the injection of lidocaine alone, combination of dexmedetomidine and lidocaine can be used for a better pain control in molar surgeries.

## 1. Introduction

Despite significant advances in pain management in dentistry, pain remains a major concern for many patients [1]. Surgical procedures for the extraction of impacted molar teeth are often associated with lots of discomfort and difficulty [2]. Since surgeons try to reduce postoperative complications, various approaches have been examined to minimize postoperative complications [3]. Pain is one of the most important complications in the extraction of molar teeth which can even cause the patients not willing to seek further dental treatment. The pain after the surgery causes discomfort, delays the resumption of daily activities, and necessitates the use of sedatives [4]. Effective pain control can help to improve outcomes of the surgery, also result in shorter hospital stays, and, on the other hand, reduce the risk of chronic pain in the patients [5].

Becoming aware of the need for a surgery evokes feelings of fear and anxiety in many patients. Sedatives can increase pain threshold, exert antianxiety effects, and ultimately influence and control patients' pain [6]. Glucocorticosteroids, long-acting local anesthetics, and nonsteroidal anti-inflammatory drugs are commonly used for this purpose [7]. In addition to traditional analgesics, i.e., propofol and lidocaine, dexmedetomidine has been recently administered in the field of anesthetics [8, 9]. Dexmedetomidine is an *α*2-adrenoreceptor agonist which is highly selective. It triggers and also maintains the sleeping state by stimulating the densest region of *α*2-receptors in the central nervous system which is located in the locus coeruleus in the brain stem. Patients can be aroused by language or stimuli after sedation and respiratory depression does not occur during the surgery [10, 11]. Owing to its mild analgesic and sedative effects, dexmedetomidine can reduce not only stress and anxiety but also blood pressure and heart rate [12]. A comparison of pain relievers revealed that dexmedetomidine had fewer complications, e.g., lower frequency of amnesia and tachycardia and lower systolic and diastolic blood pressures, and no side effects, e.g., unstable oxygen saturation and respiratory rate [13]. Due to its limited side effects and efficacy in pain relief, dexmedetomidine can play an important role in surgical procedures. So, dexmedetomidine has been used in intraoperative sedations [14]. Dexmedetomidine can be administered intravenously and via inhalation [15]. Some researchers indicated the greater pain relief effects of dexmedetomidine plus lidocaine injection [16]. Studies also suggest that adding dexmedetomidine to lidocaine for the surgery will increase the time of nerve block and decrease the action onset; meanwhile, it improves the postoperative pain control. The vital parameters also reported to be stable after the surgery and no complications were observed [17]. But these results need to be confirmed before it becomes a routine practice in the dentistry.

The objective of this study is to see the effects of adding dexmedetomidine to lidocaine which is the routine nerve blocker for the extraction of third molar tooth, by comparing the postoperative vital signs and pain in a randomized control trial in patients referring to Torabinejad Clinic, Isfahan University of Medical Sciences (Isfahan, Iran), in 2018.

## 2. Materials and Methods

This double-blind clinical trial was conducted on the patients referred to dental clinics affiliated to Isfahan University of Medical Sciences for mandibular hard tissue surgery in 2018. The patients were included if they were aged 18–40 years, were ASA I-II, had no contraindication for dexmedetomidine use (hypotension, bradycardia, sinus disorder, unstable hypertension, arousability, tachyphylaxis, and liver disorders), and signed an informed consent form. The exclusion criteria were lack of analgesia with the administered dose, maxillary 3^rd^ molar surgery, excessive fear of surgery, and not replying follow-up phone calls after surgery. All of the cases were also examined for the nature of impaction and expected difficulty level of the surgery by a senior maxillofacial surgeon based on the anatomical and radiological variables [18]. They were excluded if they were considered as very easy or very difficult.

**Figure 1:**
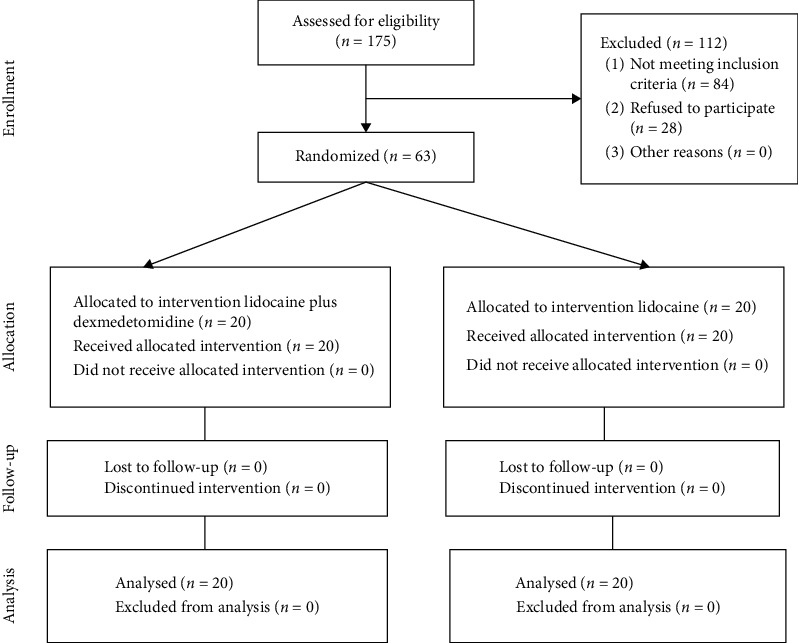
CONSORT flow diagram of the study participants.

To calculate the sample size, a preliminary study was performed on three patients in the case group and three in the control group, and the mean and standard deviation (SD) of pain score after 6 hours had been obtained to use for sample size calculation. The method of this preliminary study was the same as the main study which will be discussed later. These patients' data were not used in the main study results and analysis. In this preliminary study, the mean ± SD of the pain score after 6 hours in cases and control was 3.6 ± 2.5 and 6.35 ± 2.8, respectively. To reach the power of 80%, considering *α* = 0.05 and ratio of the cases and control = 1, the number of participants in each group was calculated as 15 by the method of sample size for comparing two means [19]. However, considering the possible loss to follow-up of 25%, 20 individuals were selected for each group. The patients were provided with information about study objectives and asked to complete an informed consent form. In order to prevent the confounding effects of age and gender, equal numbers of men and women and equal numbers of individuals from different age groups, i.e., 18–25, 25–30, and 30–40 years, were recruited into the two groups. After the enrollment, from a pool of 63 patients, a patient was selected by generating a random number between 1 and 63 using MS Excel and sent to group 1. Another patient with the same sex and age group was selected from the pool to send to group 2. If the patients with those characteristics were not in the pool, we performed the selection once again. For selecting the second patient, a number between 1 and 61 was generated and this process continued until we reached 20 patients in each group. In summary, 157 patients were assessed for the eligibility but only 20 patients were allocated to each group and analyzed. The detailed CONSORT flow diagram can be seen in Figure 1.

Two identical sets of twenty cartridges were prepared. One set was lidocaine cartridge (Exir Co., Tehran, Iran) and for making the other set, 0.15 ml of dexmedetomidine (Daru Pakhsh Co., Tehran, Iran) was added to lidocaine cartridge and the drug concentration was adjusted to 15 *μ*g. An anesthetist individually prepared the mixture. Using the same method of randomization by choosing a random number between one and two, one of the groups was assigned to one of the sets of cartridges. The surgeon and the consultants were not aware of the cartridge type. All surgeries were performed by the same surgeon. To avoid consecutive surgeries in each group, by using the same method of choosing random numbers, a number between one and forty was assigned to each person for the order of surgeries. The cartridge was also labeled by that number.

The eligible patients were first briefed about the study objectives and methods and asked to sign an informed consent form. They were then asked to complete a questionnaire containing demographic information, medical history, medicine use, and smoking. During the surgery, patient information (e.g., gender, age, and blood pressure) was recorded in a specific data collection form. A visual analog scale was also administered to measure and record patients' pain levels at the end of the surgery and 6, 12, and 24 hours later in the scale of 0 to 10, 0 corresponding no pain and 10 the worse pain. Patients were allowed to take nonsteroidal anti-inflammatory drugs (NSAIDs) or acetaminophen after the surgery and they were asked by telephone about the number of postoperative painkillers taken. The patient's satisfaction and the surgeon's assessment of the surgical process in regards to the pain control was recorded as 3-scale questionnaire, high, intermediate, and low, for the patients' satisfaction and 4-scale questionnaire, good, fair, poor, and impossible, for the surgeon's assessment of the surgical process and pain control. The patient is marked good if he or she is fully cooperative with optimum degree of sedation, marked fair if the minimal interference is necessary due to over/under sedation, marked as poor if the operation is difficult due to over/under sedation, and marked impossible if actions such as general anesthesia are required.

All patient information was recorded anonymously and the participants were ensured about the confidentiality of the collected data. The patients paid no fees for pre- and postoperative tests. Informed consent was obtained from all subjects before the intervention. The study protocol was approved by the Ethics Committee of Isfahan University of Medical Sciences (ID = 397331).

Data collected through questionnaires were coded and analyzed using Fisher's exact test, ANOVA, or Mann–Whitney U test wherever appropriate. Spearman rank correlation analysis was performed by dichotomizing the pain score values using the 75 percentiles (third quartile) of the patient's pain scores as the cutoff value, between pain score and the intervention group, using the intervention group as 1 and the control group as 0. All analyses were performed using SAS version 9.4 (SAS, Cary, NC, USA) at a significance level of *P* < 0.05. After the results have been obtained, the power of the study was determined by using free online open source calculator OpenEpi, version 3, by the method of comparing two means for the pain score between the interventional and control groups [19].

## 3. Results

From the total of 40 patients, 17 were men (42.5%) and 23 were women (57.5%). Assessment of 36 patients (901%) was good and 4 patients (10%) was fair. Satisfaction rate of 35 patients (87.5%) was high and 5 patients had moderate satisfaction.  Table 1 shows the characteristics of the patients in each group. Except pulse rate of the patients which is significantly higher in the intervention group, other vital signs are not different between the two groups.

The two groups had no significant differences in the number of painkillers used at 6, 12, and 24 h after surgery. However, pain score was significantly lower in the intervention group at the time of surgery and also 6, 12, and 24 hours after that (Table 2). The third quartile of pain score was set as the cutoff value for high and low pain. The 75% quartile of pain score for the times 0, 6, 12, and 24 was 1, 8, 6.5, and 5. The Spearman rank correlation analysis for pain score in different times based on different groups (intervention or control) can be seen in Table 2. Pain score is negatively correlated with the intervention, during 6 and 12 hours after the surgery, but loses its significant correlation 24 hours after the surgery.

By considering the mean pain score and the SD in the intervention and control groups, the power of the study during the surgery and 6, 12, and 24 hours after the surgery was 61.47%, 98.56%, 9.53%, and 87.75%.

## 4. Discussion

The findings of this study demonstrated that adding dexmedetomidine to lidocaine cartridge increased the effects of lidocaine and reduced pain scores in patients immediately and 6, 12, and 24 hours after surgery. The correlation of pain score and the intervention is negative, meaning adding dexmedetomidine to lidocaine cartridge correlates with decreasing the pain score in the patients. This significant correlation had been observed during the surgery as well as 6 and 12 hours after that. After 24 hours although the patients had lower pain scores, the difference between two groups was not significant. We also found out that, although the intervention group used fewer painkillers, there were no significant differences between the two groups in terms of the mean number of painkillers used. So, the higher pain score did not result in taking significantly more painkillers.

Molar surgery and the associated pain lead to various complications including decreased quality of daily activities, excessive use of sedatives, and increased risk of polypharmacy [20]. Studies on the reduction of these complications and pain relief after dental surgery have focused on the use of steroidal drugs, such as glucocorticosteroids, and nonsteroidal anti-inflammatory drugs [17]. However, the use of topical and anesthetic treatments is also of paramount importance [21]. Lidocaine is an important agent used for pain relief after dental surgery [22]. Moreover, as a pain reliever and *α*2-adrenoreceptor agonist, dexmedetomidine plays a key role in reducing postoperative complications. The drug's mechanism of action is by inhibiting epinephrine and norepinephrine release and thus decreasing patient stress through eliminating the feelings of confrontation and escape [11, 23].

Several studies have evaluated the effects of dexmedetomidine and lidocaine on pain relief. In a study on healthy individuals in 2014, Yamane et al. [16] showed that dexmedetomidine injection increased pain threshold and decreased feeling of pain. They observed the highest increase in pain threshold at 10 minutes after injection. Furthermore, an increase in pain threshold and, thus pain relief, was maximized 20 minutes after the administration of lidocaine + dexmedetomidine. Dexmedetomidine administration did not alter levels of blood pressure, heart rate, and drowsiness in healthy individuals. Although all the patients in our study had normal and stable vital signs, the pulse rate of the patients was significantly higher in the intervention group and remained high after the surgery.

A double-blind study by Shetty et al. in 2016 [21] evaluated the levels of consciousness in 15 patients undergoing third molar extraction surgery. The results showed that pain severity and consciousness levels were significantly lower in those receiving dexmedetomidine than in the placebo group. In contrast, in 2016, Mishra et al. [13] reported that dexmedetomidine administration reduced amnesia and systolic and diastolic blood pressure, but failed to relieve pain. In a clinical trial in 2007, Cheung et al. [24] compared the effects of dexmedetomidine and midazolam on third molar surgery. They noticed that dexmedetomidine injection lowered blood pressure and heart rate compared to midazolam but had no significant pain relief effects. In line with some previous studies, the overall results of this study indicated that dexmedetomidine injection relieved pain and, hence, reduces the use of painkillers to some extent. Some studies, however, showed that dexmedetomidine injection did not significantly affect pain levels. Although our observations are consistent with those of some previous studies, further clinical trials are recommended to confirm these results.

Our study had some limitations including a relatively small sample size (due to lack of access to further facilities and time and space limitations), although the power of our study was very high, especially 6, 12, and 24 hours after the surgery, regarding the pain scores. Using only one drug dose (rather than various doses) was another limitation of this study. Given more efficiencies reported in the coadministration of other pain relievers, further studies with larger sample sizes are recommended to use combinations of dexmedetomidine and other drugs. An advantage of this study was the control of factors affecting the final results, which can provide more realistic and reliable outcomes.

## 5. Conclusions

In patients undergoing molar surgery, administration of a combination of dexmedetomidine and lidocaine reduced pain scores to a significantly greater extent compared to lidocaine alone and decreased the number of painkillers compared to the control group. Therefore, a combination of dexmedetomidine-lidocaine is recommended for further pain relief and reducing the use of analgesics in patients undergoing such procedures.

## Figures and Tables

**Table 1:** Patient characteristics in the intervention and control group.

Variable		Intervention group (dexmedetomidine + lidocaine)	Control group (lidocaine)	*P* value (test)
Age (year)		27.15 ± 5.54	27.05 ± 5.51	0.93
Gender	Female	12 (60%)	11 (55%)	0.50
	Male	8 (40%)	**9 (45%)**	

Assessment	Good	20 (100%)	16 (80%)	0.10
	Fair	0 (0%)	4 (20%)	

Satisfaction	High	19 (95%)	16 (80%)	0.34
	Intermediate	1 (5%)	4 (20%)	

Age (year)		27.15 ± 5.54	27.05 ± 5.51	0.93
SBP before injection		120.5 ± 10.99	113 ± 13.41	0.06
SBP after injection		122.5 ± 11.18	119 ± 13.37	0.37
SBP after surgery		120.5 ± 10.99	117 ± 13.41	0.37
DBP before injection		75 ± .5±6.86	72 ± 9.51	0.19
DBP after injection		77 ± 9.23	74.5 ± 8.25	0.37
DBP after surgery		75 ± 6.09	73 ± 7.32	0.35
O2 before injection		97.15 ± 1.95	97.8 ± 1.15	0.20
O2 after injection		96.85 ± 2.08	97.45 ± 1.19	0.27
O2 after surgery		96.55 ± 1.87	97.20 ± 1.05	0.18
PR before injection		86.75 ± 7.41	84.40 ± 9.32	0.38
PR after injection		90.50 ± 7.38	84.95 ± 8.16	**0.03**
PR after surgery		89.55 ± 7.97	84.55 ± 6.56	**0.03**

Assessment: surgeons' assessment of patient's pain control during surgery. Satisfaction: patients' satisfaction of pain control during surgery; SBP, systolic blood pressure; DBP, diastolic blood pressure; O_2,_ blood oxygen saturation; PR, pulse rate. *P* < 0.055

**Table 2:** The mean pain scores and number of painkillers used in the two groups at different times.

Variable	Time	Intervention	Control	*P* value	Spearman (r)	Spearman *P* value
Pain scores (mean ± SD)	0	0.25 ± 0.44	0.8 ± 1.00	0.031	−0.33	0.03
	6	4.40 ± 2.50	7.55 ± 2.03	<0.001	−0.37	0.01
	12	2.80 ± 2.52	5.50 ± 2.39	0.003	−0.46	<0.01
	24	1.60 ± 2.37	4.00 ± 2.49	0.003	−0.17	0.26

Painkillers used (number ± SD)	6	1.4 ± 0.75	1.6 ± 0.75	0.313	—	—
	12	0.75 ± 0.55	1.1 ± 0.71	0.109	—	—
	24	0.45 ± 0.60	0.70 ± 0.80	0.348	—	—

Time, time after the surgery (hours); intervention, intervention group (dexmedetomidine + lidocaine); control, control group (lidocaine); Spearman (r): Spearman correlation (r).

## Data Availability

The datasets used and/or analyzed during the current study are available from the corresponding author on reasonable request.
